# Epidural Dexmedetomidine Reduces the Requirement of Propofol during Total Intravenous Anaesthesia and Improves Analgesia after Surgery in Patients undergoing Open Thoracic Surgery

**DOI:** 10.1038/s41598-017-04382-5

**Published:** 2017-06-21

**Authors:** Xianzhang Zeng, Jingjing Jiang, Lingling Yang, Wengang Ding

**Affiliations:** Department of Anaesthesiology, Second Hospital of Harbin Medical University, 246 Xuefu Road, Harbin, 150001 Heilongjiang China

## Abstract

The aim of this study was to assess the systemic and analgesic effects of epidural dexmedetomidine in thoracic epidural anaesthesia (TEA) combined with total intravenous anaesthesia during thoracic surgery. Seventy-one patients undergoing open thoracotomy were included in this study and randomly divided into three groups: Control group (Group C): patients received TEA with levobupivacaine alone and were intravenously infused with saline; Epidural group (Group E): patients received TEA with levobupivacaine and dexmedetomidine, and were intravenously infused with saline; Intravenous group (group V): patients received TEA with levobupivacaine alone and were intravenously infused with dexmedetomidine. The doses of propofol used in the induction and maintenance of general anaesthesia, cardiovascular response, dose and first time of postoperative analgesia and verbal rating scale were recorded. The induction and maintenance were significantly lower in the Groups E and V. Verbal rating scale and postoperative analgesic requirements were significantly lower in Group E than in Groups C and V. Patients in Group C had more severe cardiovascular responses, as compared with Groups E and V. Epidural administration of dexmedetomidine reduced the induction and maintenance of propofol, and inhibited the cardiovascular response after intubation and extubation. Moreover, epidural dexmedetomidine provided better analgesia after open thoracotomy.

## Introduction

Intraoperative hypoxemia and severe postoperative pain are two important factors considered by anaesthesiologists in anaesthetic management during thoracic surgery. Hypoxemia, which is usually caused by intrapulmonary shunting, leads to a decrease in the partial pressure of oxygen (PaO_2_) and oxygen saturation (SaO_2_). Hypoxic pulmonary vasoconstriction is an important protective mechanism to decrease the risk of intrapulmonary shunting^1^. Severe postoperative pain will impair pulmonary function, deep breathing and effective coughing^2^, thereby increasing the risk of perioperative morbidity^3^. Compared with inhalation anaesthetic, propofol reduces the inhibition of hypoxic pulmonary vasoconstriction^4^. In addition, thoracic epidural anaesthesia is one of the most effective methods to achieve post-thoracotomy pain relief^5^. Therefore, total intravenous anaesthesia combined with TEA is a preferred anaesthetic technique for open thoracotomy.

Over the past few years, many studies demonstrated that intravenous dexmedetomidine can reduce the dosage of inhalation and intravenous administration of anaesthetics and decrease the risk of intrapulmonary shunting to achieve stable intraoperative hemodynamics^4–7^. Yet, to date, there is little published data addressing the possible beneficial effects of epidural dexmedetomidine when combined with total intravenous anaesthesia in thoracic surgery. Meanwhile, other studies found that dexmedetomidine conveys some analgesic effects with few side effects^8–11^. Furthermore, there are some analgesic advantages to neuraxial administration of dexmedetomidine, as compared with systemic administration^12–14^. Our previous studies also confirmed that epidural administration of low-dose dexmedetomidine can improve the effects of thoracic anaesthesia^10, 11^.

In this study, the systemic and analgesic effects of epidural dexmedetomidine in thoracic epidural anaesthesia combined with total intravenous anaesthesia during thoracic surgeries were assessed. We hypothesized that epidural administration of dexmedetomidine provides similar beneficial systemic effects and better postoperative pain relief than intravenous administration. The primary endpoint was the requirement of propofol doses during induction (Induction Dose, ID). The secondary outcome measures included the postoperative pain intensities, as assessed with a verbal rating scale (VRS) at rest and after coughing, the requirement of propofol doses during the maintenance period of anaesthesia (Maintenance Dose, MD), cardiovascular responses during endotracheal intubation and the risk of intrapulmonary shunting.

## Results

**Figure 1:**
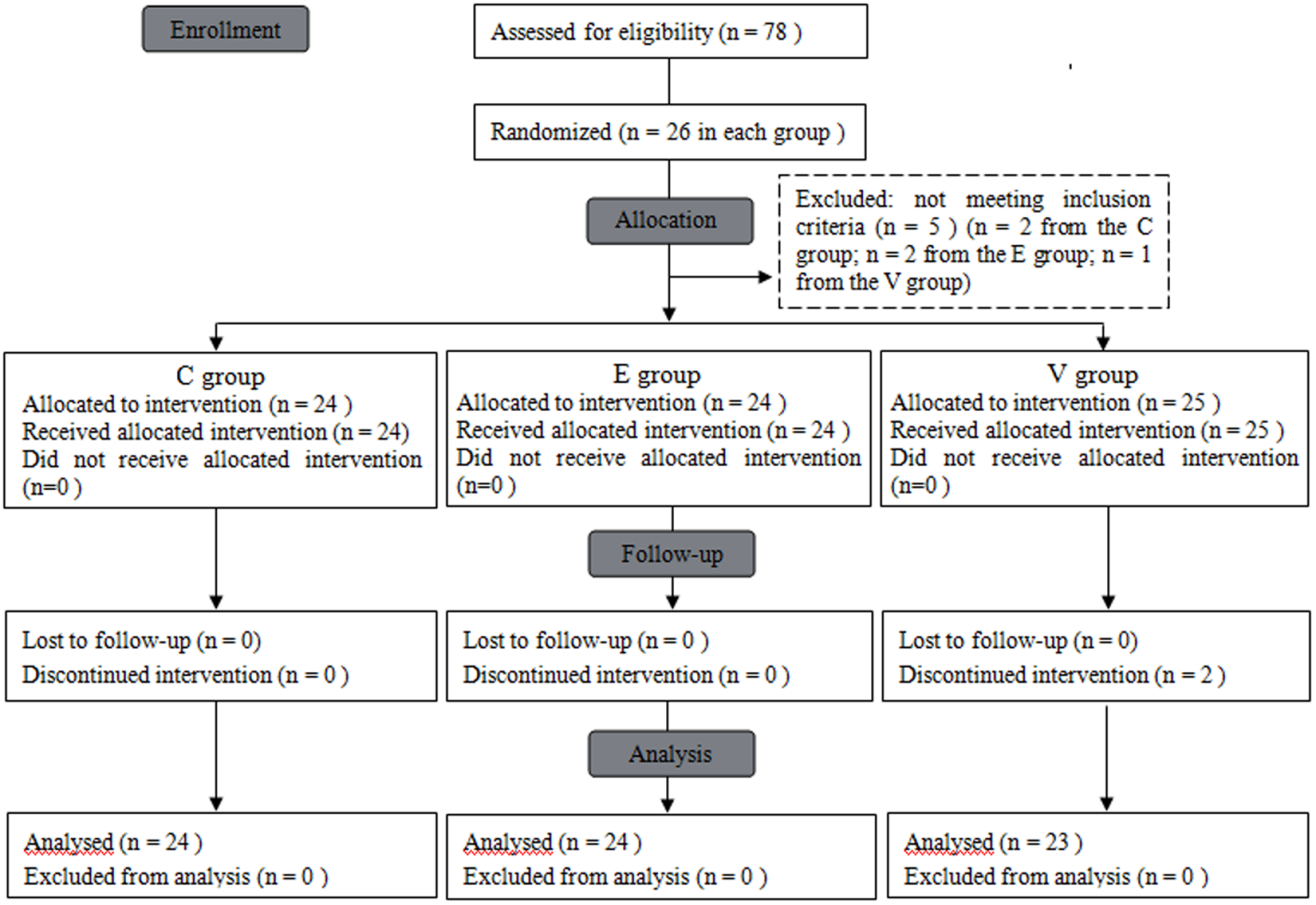
The study design and the flow of subjects.

Of the 78 patients initially randomized into the study, five (n = 2 from Group C; n = 2 from Group E; n = 1 from Group V) were withdrawn because of contraindications to regional techniques. Two patients in Group V were excluded because of intrathoracic bleeding within the first 3 days after surgery, which required intervention. Therefore, 71 patients completed the study (Fig. 1).

**Figure 2:**
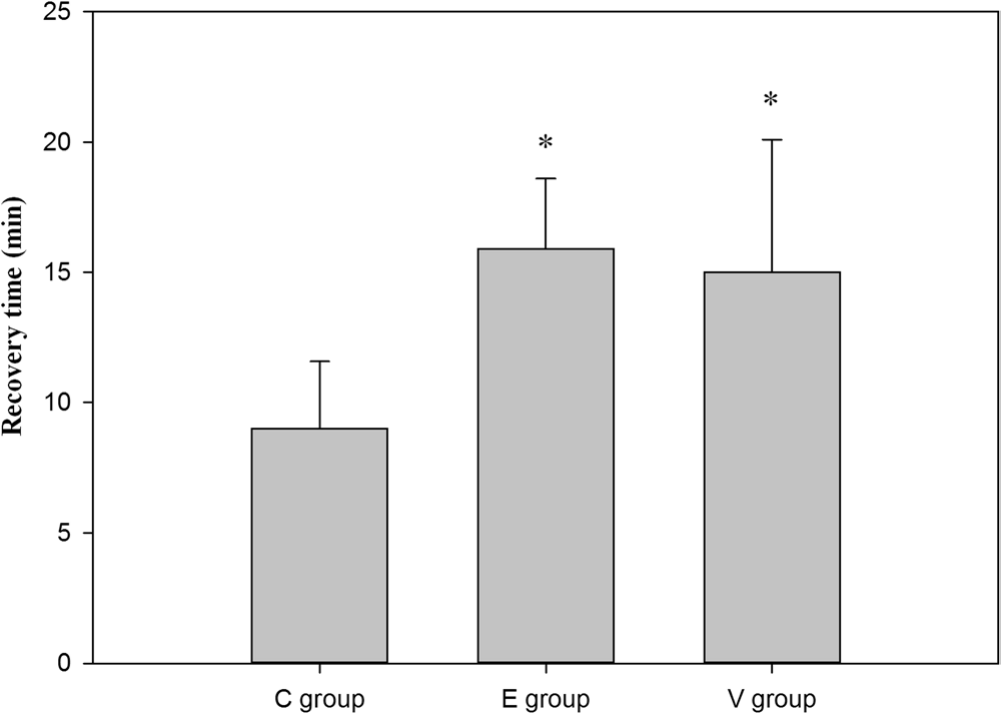
Propofol doses for the recovery time after cessation of propofol infusion to the BIS level of 80 (mean and SD). *: compared with Group C, *p* < 0.05.

There were no significant differences in patient or surgery characteristics among the groups (Table 1). The ID (mg/kg) and MD (mg/kg/min) were significantly higher in Group C (ID: 1.67 ± 0.34; MD: 0.12 ± 0.05) than in Group E (ID: 1.3 ± 0.32, *p* < 0.001; MD: 0.09 ± 0.04, *p* = 0.001) and Group V (ID: 1.33 ± 0.35, *p* *=* 0.012; MD: 0.1 ± 0.02, *p* *=* 0.04). There was no significant difference in ID and MD between Groups E and V. The time to reach the bispectral index (BIS) level of 80 during the anesthesia emergence period was significantly shorter in Group C than in Groups E and V (*p* < 0.01) (Fig. 2), while there was no significant difference between Groups E and V (*p* = 0.533).

**Table 1:** Baseline characteristics and surgical aspects of the included patients in all groups.

	Group C (n = 24)	Group E (n = 24)	Group V(n = 23)	*p*
Age (year)	57 ± 8	55 ± 12	58 ± 12	0.711
Male Sex (%)	41.7	45.8	39.1	0.897
Body mass index (kg/m^2^)	22.9 ± 2.7	22.3 ± 3.2	21.7 ± 3.6	0.392
ASA physical status (n)				0.854
I	11	11	9	
II	10	12	12	
III	3	1	2	
Lung function (L)				
FEV1	2.47 ± 0.74	2.59 ± 0.71	2.61 ± 0.76	0.788
FVC	3.09 ± 0.87	3.29 ± 0.85	3.17 ± 0.84	0.751
SpO2 on room air	96.7 ± 1.5	96.3 ± 2.4	96.5 ± 1.7	0.649
Types of surgery (n)				0.523
Segment resection	2	3	2	
Lobectomy	16	13	10	
Esophagectomy	6	8	11	
History of sedatives and analgesics (%)	29.2	20.8	39.1	0.389
Duration of surgery (min)	161 ± 39	159 ± 56	151 ± 49	0.973
Doses of aramine (mg)	2.8 ± 1.9	2.4 ± 1.3	2.4 ± 0.7	0.561
The incidence of bradycardia (%)	8.3	12.5	13.0	0.903
The time to the first pethidine (min)	153 ± 80	854 ± 459	528 ± 371	<0.001
The total dose of pethidine (mg)	162 ± 106	88 ± 80	148 ± 95	0.018

**Figure 3:**
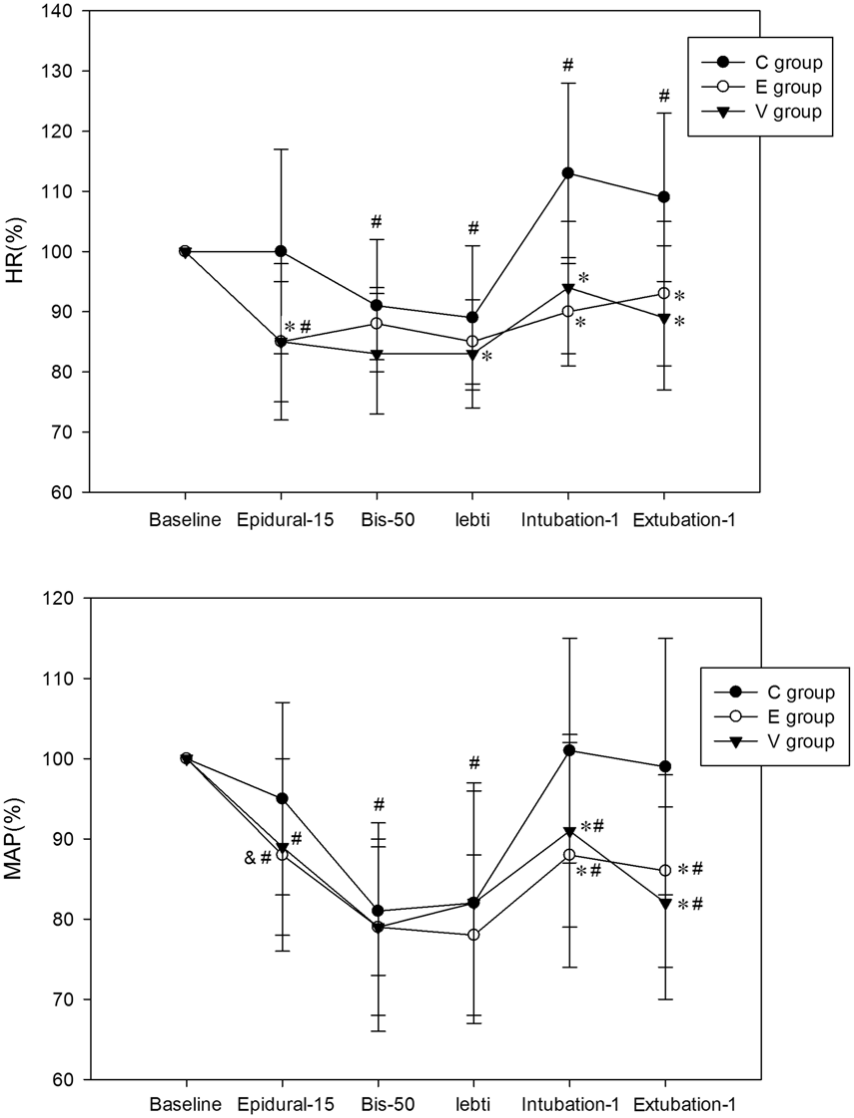
Changes in HR and MAP in the Groups C, E and V. HR, heart rate; MAP, mean arterial pressure; Epidural-15, 15 min after epidural or intravenous infusion of dexmedetomidine; BIS-50, at the time of the BIS level of 50; Lebti, the time before tracheal intubation; Intubation-1, 1 min after intubation; Extubation-1, 1 min after extubation. Measurements were recorded at baseline, Epidural-15, BIS-50, Lebti, Intubation-1, and Extubation-1. * compared with Group C, p < 0.01; # compared with baseline, p < 0.01; & compared with Group C, p < 0.05.

In Groups E and V, heart rate (*p* < 0.01) and mean arterial pressure (*p* < 0.01) were significantly decreased, as compared with baseline values at 15 min after epidural or intravenous infusion of dexmedetomidine, BIS level of 50, the time before tracheal intubation, 1 min after intubation and 1 min after extubation. In Group C, heart rate (*p* < 0.01) and mean arterial pressure (*p* < 0.01) were significantly decreased at the time of BIS level of 50 and before tracheal intubation compared with baseline values, while heart rate increased significantly at 1 min after intubation and 1 min after extubation (*p* < 0.01), but there was no increase in mean arterial pressure. Heart rate was significantly lower in Groups E and V, as compared with Group C at 15 min after epidural or intravenous infusion of dexmedetomidine, 1 min after intubation and 1 min after extubation (*p* < 0.01). In Group E, mean arterial pressure was significantly decreased at 15 min after epidural or intravenous infusion of dexmedetomidine as compared with Group C (*p* < 0.05). And mean arterial pressure was decreased at 1 min after intubation and 1 min after extubation in Group E and V, as compared with Group C (*p* < 0.01). For heart rate and mean arterial pressure, there were no significant differences at any time point between Groups E and V. (Fig. 3). The increased concentration of catecholamine was significantly higher in Group C than in Groups E and V (*p* < 0.05) (Table 2)

**Table 2:** The increases of catecholamine value, intrapulmonary shunt fraction and the concentration of dexmedetomidine in all groups.

	C group (n = 24)	E group (n = 24)	V group (n = 23)	*P*
The increase of catecholamine value (%)	160.7 ± 29.8	136.7 ± 21.4	140.7 ± 19.0	0.025
The value at baseline (ng/L)	123.8 ± 21.7	125.6 ± 15.4	128.2 ± 18.3	0.82
The value at 1 min after intubation (ng/L)	193.3 ± 15.9	169.0 ± 10.1	178.1 ± 16.7	<0.001
Intrapulmonary shunt fraction (%)				
TLV-10	6.6 ± 1.3	7.4 ± 2	6.8 ± 1.4	0.446
OLV-20	27.2 ± 4.2	26.4 ± 2.2	26.3 ± 1.7	0.663
Concentration of dexmedetomidine (ng/ml)				
Epidural-15	0.00	0.46 ± 0.12	0.44 ± 0.14	0.653
TLV-10	0.0	0.41 ± 0.13	0.39 ± 0.11	0.533
OLV-20	0.0	0.26 ± 0.11	0.27 ± 0.10	0.808

Epidural-15, 15 min after epidural or intravenous infusion of dexmedetomidine; TLV-10, 10 min after two-lung ventilation; OLV-20, 20 min after one-lung ventilation.

**Figure 4:**
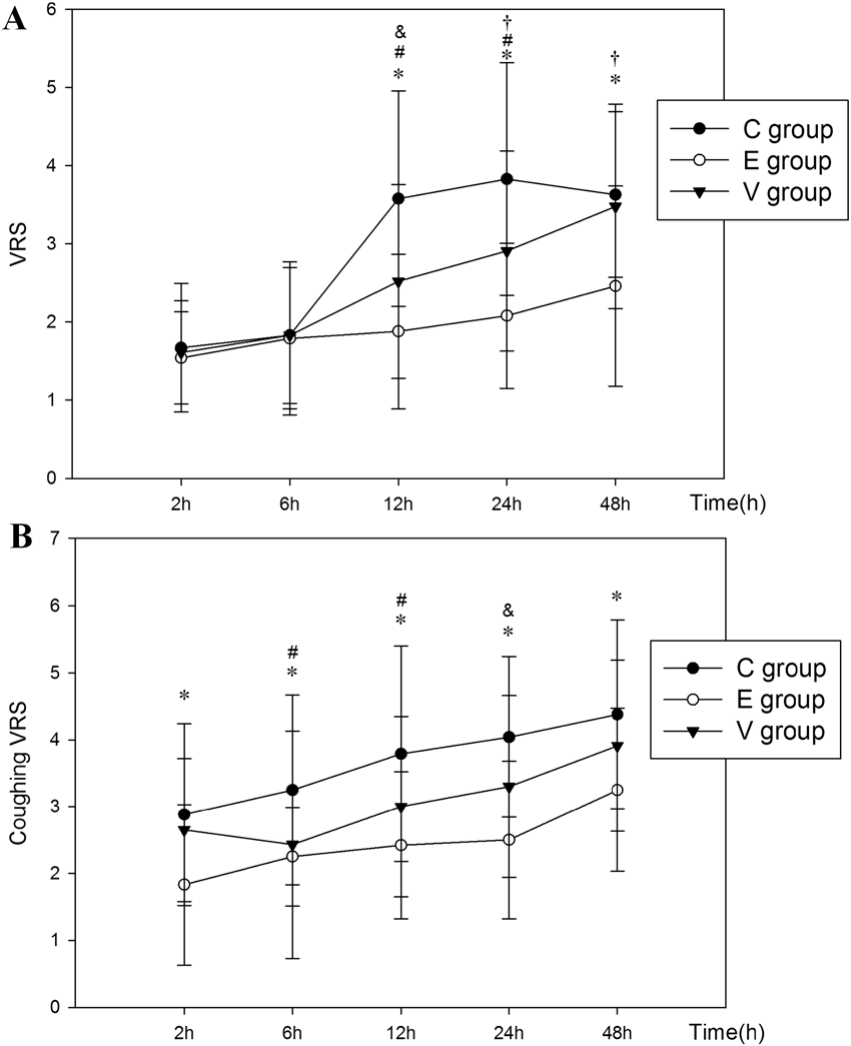
Changes in resting VRS (a) and coughing VRS (B) (mean and SD) in Group C, Group E and Group V. Measurements were recorded at 2, 6, 12, 24 and 48 h after surgery. Group E vs. Group C, *p < 0.01; Group C vs. Group V, ^#^ *p* < 0.05; Group E vs. Group V. ^&^ *p* < 0.05 and ^†^ *p* < 0.01.

Resting VRS was significantly lower in Group E than in Group C at 12, 24 and 48 h (*p* < 0.01) and in Group V at 12 h (*p* < 0.05), 24 h (*p* < 0.01) and 48 h (*p* < 0.01) after surgery. But in Group V, resting VRS was lower only at 12 and 24 h after surgery, as compared with Group C (*p* < 0.05) (Fig. 4A). During coughing, VRS increased at the majority of time points, although coughing VRS was still significantly lower in Group E than in Group C at all postoperative time points (*p* < 0.01), while there were only significant differences at 6 and 12 h after surgery between Groups V and C (*p* < 0.05). The coughing VRS was significantly decreased in Group E, as compared with Group V at 24 h after surgery (*p* < 0.05) (Fig. 4B). At the time of administration of the first analgesic (pethidine), there were significant differences between all groups (*p* < 0.01). The total dose of pethidine was significantly lower in Group E than in Group C (*p* < 0.01) and Group V (*p* < 0.05), but there was no difference between the Groups C and V (*p* = 0.594) (Table 1).

No significant differences were observed among groups in either the intrapulmonary shunt fraction or the concentration of dexmedetomidine at the observed time points. There were also no significant differences in the doses of aramine and the incidence of bradycardia, upper or lower boundary of blocking levels, and the incidence of side effects related to the analgesics among the groups. No patient developed neurologic deficits and intraoperative awareness (Tables 1, 2 and 3).

**Table 3:** The comparison of blocking levels and postoperative side effects observed in all groups.

	Group C	Group E	Group V	*P*
	(n = 24)	(n = 24)	(n = 23)	
Upper boundary of blocking levels				0.299
T1	2	5	5	
T2	20	19	18	
T3	2	0	0	
Lower boundary of blocking levels				0.242
T7	5	0	3	
T8	12	17	14	
T9	6	4	5	
T10	1	3	1	
Side effects				
Nausea and vomiting	4	0	2	0.113
Skin itching	3	1	1	0.609
Hypotension	4	2	2	0.717
Bradycardia	1	0	0	0.333
Rigors	2	0	1	0.381
Neurologic deficits	0	0	0	—
Intraoperative awareness	0	0	0	—

## Discussion

The results of this study showed that epidural and intravenous administration of dexmedetomidine decreased the requirement of propofol for the induction and maintenance of general anaesthesia and did not produce serious intraoperitive change of the hemodynamics. Moreover, epidural administration of dexmedetomidine provided better postoperative pain control after open thoracotomy, as compared with Groups V and C. Together, these results confirmed our previous hypothesis that the beneficial effects of epidural administration of dexmedetomidine indicate that thoracic epidural anaesthesia combined with total intravenous anaesthesia is suitable for open thoracotomy. However, neither epidural nor intravenous administration of dexmedetomidine reduced the incidence of intrapulmonary shunting.

Many studies have demonstrated that intravenous dexmedetomidine has some beneficial effects, such as a reduction in the dose of general anaesthetics^13, 14^ and more stable hemodynamics during general anaesthesia^16^. This may be due to the sedative effect of dexmedetomidine, which is mediated through the locus coeruleus in the brain stem, where dexmedetomidine decreases sympathetic outflow and increases parasympathetic outflow^17, 18^. The results of this study are consist with those of these previous studies. Meanwhile, epidural and intravenous administration of dexmedetomidine achieved similar systemic effects. In the present study, the decreased concentration of catecholamine at 1 min after intubation in Groups V and E was also consistent with the changes in mean arterial pressure and heart rate. Although the mechanism remains unclear, these results may be related to the similar blood concentration of dexmedetomidine. Although epidural dexmedetomidine may also act in the spinal cord^19^ and appear in the cerebrospinal fluid^10^, we speculated that the systemic effects of epidural dexmedetomidine in this study was due to “systemic absorption” after intrathecal administration of local anaesthetic^20, 21^. The similar blood concentration of dexmedetomidine, which was tested at 15 min after epidural or intravenous administration, support this speculation.

In the last few years, many studies had found that dexmedetomidine can be administered perineurally in combination with local anaesthetics to block nerve conduction^22–24^ and intravenously or intrathecally during spinal or epidural anaesthesia^8, 25, 26^. These studies found that dexmedetomidine can potentiate the analgesic effects of local anaesthetics with few side effects. The results of postoperative pain control in this study were consistent with those reported in previous studies, indicating that epidural dexmedetomidine with morphine can result in better analgesia, as compared with intravenous dexmedetomidine combined with epidural morphine or epidural morphine alone. Two factors may contribute to the mechanism of this effect. The first may be related to the improved analgesic effect of epidural morphine. Because of the high affinity binding with α2C adrenoceptors^27^, dexmedetomidine analgesia may have a synergistic effect with spinal cord opioid analgesia^19^. All patients in this study received epidural morphine for postoperative analgesia, thus the analgesic effect mediated by epidural dexmedetomidine may reflect a synergistic effect with morphine. In addition, epidural dexmedetomidine can also produce an analgesic effect. Because of lipophilicity, dexmedetomidine may be rapidly absorbed into the cerebrospinal fluid and provide a central analgesic effect^10^. On the other hand, the spinal, supraspinal, and peripheral actions may also contribute the epidural dexmedetomidine analgesic effects. Studies demonstrated that the dorsal horn nociceptive neurons and peptides released from spinal cord slices are inhibited by adrenergic agonists^19^. Therefore, the analgesia effect of epidural dexmedetomidine may be related in direct and indirect ways.

One study showed that the intravenous infusion of dexmedetomidine at 0.7 μg/kg/h decreased the risk of intrapulmonary shunting and moderated change in PaO2, indicating potential usefulness in the management of one-lung ventilation^13^. Another study indicated that epidural administration of dexmedetomidine at 1 μg/kg can improve intraoperative oxygenation^28^. However, the results of this study did not find that intravenous or epidural administration of dexmedetomidine at 0.5 μg/kg can influence the risk of intrapulmonary shunting. We speculated that these results may be related to two aspects. First, inhalation anaesthesia, such as with isoflurane, is known to inhibit the hypoxic pulmonary vasoconstriction, which aggravates intrapulmonary shunting. The decreased incidence of intrapulmonary shunting, as reported in two previous studies, may be due to the reduced dose of inhalation anaesthetic. Propofol did not inhibit hypoxic pulmonary vasoconstriction as much as the use of inhalation anaesthetic^4^. Therefore, the reduced dose of propofol induced by dexmedetomidine in this study did not significantly influence the incidence of intrapulmonary shunting during one-lung ventilation. On the other hand, one study demonstrated that dexmedetomidine can activate nitric oxide production^29^ which can decrease the regional pulmonary vascular resistance of the ventilated lung area. Therefore, the incidence of intrapulmonary shunting can be decreased and arterial oxygenation improved^30^. We speculated that the dose of dexmedetomidine used in this study may have been too small to induce a sufficient volume of nitric oxide. There was no significant difference in the incidence of intrapulmonary shunting between Groups V and E, which may have been related to the similar blood concentration of dexmedetomidine at the time of 10 min after two-lung ventilation and 20 min after one-lung ventilation.

Other studies have reported that local anaesthetics have a sedative effect when administered spinally and epidurally^31–33^. This effect of local anaesthetics may blanket the effect of epidural dexmedetomidine on the requirement of propofol. However, we used the same local anaesthetic in all groups. Therefore, we think the bias was minimal. In addition, another study indicated that the sensorial levels of epidural anaesthesia, but not the volume of local anaesthetic, influenced the propofol requirement during induction of general anaesthesia^21^. The sensorial block levels of epidural anaesthesia in this study were similar. Therefore, the reduced requirement of propofol in this study may have been due to the use of dexmedetomidine.

There were no neurologic deficits in any patients in this study, which confirmed the safety of epidural dexmedetomidine, consistent with the results of previous studies that used local injection and reported no neurologic deficits^6–9, 23, 34–37^. A recent animal study also supported this point of view in which epidural dexmedetomidine showed protective effects against neural cell death induced by lidocaine and had no obvious pathologic impact on the spinal cord^38^. Therefore, we speculated that epidural dexmedetomidine was safe and efficacious, although future studies with larger samples are still needed.

In conclusion, the results of this study partly confirmed our previous hypothesis that epidural dexmedetomidine could reduce the ID and MD of propofol, and inhibit the cardiovascular response after intubation and extubation of the double-lumen tube as well as intravenous administration. Moreover, epidural administration of dexmedetomidine provided better analgesia after open thoracotomy. However, both routes of administration of dexmedetomidine did not influence the incidence of pulmonary arteriovenous shunting and, therefore was insufficient to ameliorate hypoxemia during one-lung ventilation, and may lead to the delayed recovery of total intravenous anaesthesia.

## Subjects and Methods

The protocol of this prospective, randomised, double blind, controlled study was approved by the ethics committee of Harbin Medical University, Harbin, China (approval number: HMUIRB20150016) and conducted in accordance with the tenets of the Declaration of Helsinki. The study was registered in the Chinese Clinical Trial Registry on 10 July 2015 (registration number: ChiCTR-IPR-15006727). Patients were recruited between August 2015 and May 2016, and all submitted written informed consent. Patients aged between 18 and 70 years who met the American Society of Anaesthesiology Physical Status I – III criteria and underwent open thoracotomy were included in this study. Exclusion criteria were neurologic or psychiatric illness, diabetes, pregnancy, renal or hepatic insufficiency, allergy to local anaesthetics, coagulation abnormalities or anticoagulant therapy.

After computer-generated randomization, patients were randomly assigned to one of three groups: a control group (Group C), in which patients received thoracic epidural anaesthesia with levobupivacaine alone and were infused intravenously with saline; an epidural group (Group E), in which patients received thoracic epidural anaesthesia with levobupivacaine and dexmedetomidine, and were infused intravenously with saline; and an intravenous group (Group V), in which patients received thoracic epidural anaesthesia with levobupivacaine alone, and were infused intravenously with dexmedetomidine. All patients received intravenous midazolam (0.02 mg/kg) and sufentanil (0.08 μg/kg) 5 min before the measurement at baseline Routine monitoring included electrocardiography, heart rate, pulse oximetery and the bispectral index. Radial artery catheters were placed for invasive blood pressure monitoring and arterial blood gas sampling. After local anaesthetic infiltration, central venous catheters were inserted into the right jugular vein for infusion and approximate mixed venous blood gas sampling. The depth of the central venous catheter was calculated according to the distance between the puncture point and the thoracic lock joint, and the distance between the thoracic lock joint and the angulus Ludovici.

The epidural catheter was placed in the T5/6 interspace in all patients using a midline approach. Loss of resistance to saline was used to identify the epidural space and a test dose of 2% lidocaine with 3.0 ml of 1:200,000 adrenaline to detect intrathecal or intravascular misplacement. After the test, 7 ml of 0.375% levobupivacaine combined with or without dexmedetomidine (0.5 μg/kg) was administered. Patients in Group V received intravenous dexmedetomidine (0.5 μg/kg) for 10 min before administration of epidural local anaesthetic. At 15 min after epidural administration, the sensorial block was recorded and each patient received total intravenous anaesthesia.

After preoxygenation for 3 min, propofol was delivered at a rate of 250 μg/kg/min until the BIS was 50 for 5 s. No stimulation was performed during the anaesthesia induction period. Another blinded anaesthesiologist observed the BIS score and determined the end point of titration. The requirement for propofol was recorded. After intravenous administration of vecuronium (0.1 mg/kg) and sufentanil 0.4 (μg/kg), a left-side double-lumen tube (Broncho-Catht, Mallinckrodt, Athlone, Ireland) was inserted. The position of the tube was assured by fiberoptic bronchoscopy after intubation in the lateral decubitus position. The mean arterial pressure and heart rate were recorded at baseline, 15 min after epidural or intravenous infusion of dexmedetomidine, BIS level of 50, laryngoscopic examination before tracheal intubation, 1 min after intubation and 1 min after extubation. The mean arterial pressure and heart rate are expressed as percentages relative to baseline values.

After intubation, propofol was titrated at a rate of 10 mg/kg/h to maintain the BIS score between 40 and 60. The infusion rate was changed by 1 mg/kg/h when the BIS score was out of these limits for 10 s. The doses of propofol were recorded in milligram per kilogram per hour during surgery. Vecuronium (0.1 mg/kg/h) was used to maintain muscle relaxation. Infusion of remifentanil (5 μg/kg/h) was started after intubation until skin closure and propofol infusion was stopped at this time point and the time to reach a BIS level of 80 was recorded. Patients in Group V received intravenous dexmedetomidine (0.3 μg/kg/h) and epidural infusion of 0.375% levobupivacaine at 5 ml/h. Patients in Groups E and C received intravenous infusion of the same volume of saline and epidural infusion of 0.375% levobupivacaine at 5 ml/h combined with or without dexmedetomidine (0.3 μg/kg/h).

Bradycardia (defined as heart rate < 50 bpm) and hypotension (defined as a decrease in mean arterial pressure of >30% of the baseline value) were treated with intravenous atropine (0.5 mg) or aramine (0.25 mg). The aramine doses and the incidence of bradycardia were recorded.

Central venous blood samples (3 ml) were obtained at 15 min after epidural or intravenous infusion of dexmedetomidine and 10 min after two-lung ventilation and 20 min after one-lung ventilation to detect the dexmedetomidine concentration by ultra-performance liquid chromatography (Agilent 1290 Infinity LC System, Agilent Technologies Santa Clara, CA, USA) tandem mass spectrometry (Agilent 6430 Triple Quadrupole LC/MS system) equipped with an electrospray source. An additional 2 ml of blood were saved after central venous catheterization as baseline and at 1 min after intubation to test plasma concentrations of catecholamine using a commercially available quantitative sandwich enzyme-linked immunosorbent assay kit for human catecholamine (IBL International GmbH, Hamburg, Germany). All blood samples were centrifuged immediately at 1000 *g* for 15 min at 4 °C and stored at −20 °C before assayed. The catecholamine value was expressed as the percentage of the concentration at 1 min after intubation relative to the baseline. The intrapulmonary shunt fraction (Qs/Qt) at 10 min after two-lung ventilation and 20 min after one-lung ventilation was calculated using a standard formula.

At the end of surgery, all patients were given epidural morphine (0.04 mg/kg) followed by intravenous flurbiprofen (50 mg) every 6 h for post-operative pain control. The anaesthetist who was blinded to the grouping performed the pain severity assessment. VRS (0 to 10, 0 = no pain and 10 = worst pain imaginable) was assessed at 2, 6, 12, 24 and 48 h after surgery at rest and after coughing. The supplement analgesia was controlled by intramuscular administration of 50 mg meperidine when the VRS score exceeded 4. The time to the first supplement analgesic and total dose of supplement analgesic were recorded.

Potential analgesic side effects, including nausea and vomiting, bradycardia, skin itching and hypotension, were recorded during the postoperative period. Neurologic deficits, such as pain and numbness, were assessed at 24, 48, and 72 h and 7 days after surgery to assess the safety of epidural dexmedetomidine.

### Statistical Analysis

Statistical analyses were performed using SAS software version 9.13 (SAS Institute Inc., Cary, NC, USA). Data are expressed as the mean ± standard deviation (SD). The sample size was calculated by the ID of propofol. According to a pilot study and assuming an SD of 0.13 mg/kg and a mean of 1.56 mg/kg (Group C), 1.45 mg/kg (Group E), 1.45 mg/kg (Group V), a total of 69 patients were required in this study to achieve a power of 80% and an α of 0.05 for detection of differences among the three groups. Therefore, 78 patients (26 in each group) were enrolled in the study to compensate for possible dropouts. The Kolmogorov–Smirnov test was used to test whether the data was normally distributed. The incidence of bradycardia, sex, ASA physical status, types of surgery, history of sedatives and analgesics and postoperative side effects were analysed using the chi-squared test. The repeated measure analysis of variance was used for continuous variables (i.e., heart rate, mean arterial pressure, and VRS). The other parameters were evaluated by analysis of variance. A probability (*p*) value of < 0.05 was considered statistically significant.
